# Gilbert’s Patent Cavity Plate

**Published:** 1850-10

**Authors:** 


[*Dental News Letter.*

---

### GILBERT'S PATENT CAVITY PLATE.

The following report was made to the Pennsylvania Association of Dental Surgeons by a committee appointed to investigate the merits of Mr. Gilbert's patent plate. It will be recollected by the readers of the Recorder that we published in the December number of 1847, a communication from D. H. Porter, containing a description of this plate. We had previously tested it several times in our practice and have applied it in numerous cases since with uniform success.

Whether Mr. Gilbert was the first to make use of this central cavity (for in this the whole merit of the plan consists) or not, is a matter about which there has been some controversy; but one thing is certain, he was the first to make known and explain it to the dental public, and, as he informed the writer of this, desired nothing from those who were unwilling to recompense him for the time and expense which it had cost him, notwithstanding he has secured a patent on what he believes to be his own invention. For this liberality he is entitled to the *thanks* of the profession, if not to more substantial recompense. —*N. Y. Dental Recorder.*

From the Dental News Letter.

*To the President and Members of the Pennsylvania Association of Dental Surgeons :*

Gentlemen:—The committee appointed by you to examine into the merits of "Gilbert's Patent Cavity Plate," respectfully beg leave to report that they have attended to the duties assigned them as far as in their judgment is necessary. With reference to the priority of invention of this plate, your committee do not pretend definitely to report, inasmuch as numbers claim the originality from ten to fifteen years back; still there does not seem to be any evidence of it, except their own assertions. However, some operators have constructed a plate with a number of chambers, and consider it to have been done for the same purpose, as the single chamber claimed by Gilbert, or in other words, that the invention of one is equivalent to the invention of the other, and that substituting one chamber for any number does not entitle the modification to the credit of originality. Now inasmuch as Mr. Gilbert was the first (as far as your committee are aware) to make the Cavity Plate Public, he is entitled to the credit of the invention, so far as it subserves the public good, for we make no doubt that those who have been capable of confining it to the secrets of their own closets for fifteen years, would do so that much longer; however, your committee will leave that part of the subject, believing that his patent papers will protect him against any unjust attacks from pretending claimants.

With regard to its practical uses, your committee would report, that in a great number of cases, it has been most markedly successful, and in cases, too, where springs had been unsuccessfully applied by different operators, and they believe also that this happy result has been from the use of the "Central Cavity Plate."

This central cavity seems to be a kind of "neutral ground" or reservoir, as well for atmospheric air as the elasticity of the gum, and it is well known that the alveolar process is constantly undergoing slight absorption until it entirely disappears, and that when the plate extends over the entire surface of the hard palate, it will sooner or later impinge with more force upon it, than upon the alveolar ridge, and produce a rocking motion of the artificial teeth, a difficulty which this central cavity in a measure prevents, and as there is some elasticity in the gum at all times, unequal pressure upon any part of the operation will produce a rocking motion even of a well-fitted plain plate or a plate with cavities upon opposite sides; the hard palate will act as a pivot upon the central part of a plain plate, from the yielding character of the gums over the alveolar process, and destroy the full influence of the atmospheric pressure in many cases. This cavity plate can be applied in a large number of cases for setting a single tooth, or an indefinite number, as the accompanying specimen will illustrate.* It is an instance where two teeth have been injured by clasps, but which are now worn with entire comfort and usefulness. By an examination of a great number of cases which have been worn from four to seven months, and kept in the mouth constantly, (except while cleansing them,) no irritation of the hard palate was observable, or unpleasant consequences in any respect. In some few cases where it has not been entirely satisfactory, it seems to have resulted rather from an improper adaptation of the plate, condition of the mouth, or an inability of the patients to accommodate themselves to it, than a want of power in the plate; notwithstanding it is believed where springs have been applied, the cases are worn with greater usefulness than had the cavity not been used also.

*Resolved,* That a certificate of approval of the Centıal Cavity Plate, should be awarded Mr. Gilbert by this Society.

Signed, J. D. White, M. D.
S. T. Beale, M. D. } Committee.
Ely Parry, M. D.

The resolution reported was approved, and received the signatures of the officers of the Society.

*We saw the plate above referred to.—Ed.

